# Dimethyl 2,2′-[butane-1,4-diylbis(sul­fanediyl)]dibenzoate

**DOI:** 10.1107/S1600536809042846

**Published:** 2009-10-23

**Authors:** Iván Brito, Alejandro Cárdenas, Joselyn Albanez, Michael Bolte, Matías López-Rodríguez

**Affiliations:** a Departamento de Química, Facultad de Ciencias Básicas, Universidad de Antofagasta, Casilla 170, Antofagasta - Chile; b Departamento de Física, Facultad de Ciencias Básicas, Universidad de Antofagasta, Casilla 170, Antofagasta-Chile; c Institut für Anorganische Chemie der Goethe-Universität Frankfurt, Max-von-Laue-Str. 7, D-60438 Frankfurt am Main, Germany; d Instituto de Bio-Orgánica ’Antonio González’, Universidad de La Laguna, Astrofísico Francisco, Sánchez N°2, La Laguna, Tenerife, Spain.

## Abstract

The title compound, C_20_H_22_O_4_S_2_, was synthesized by the reaction of 1,4-dibromo­butene with methyl thio­salicylate. The aliphatic segment of this ligand is in an all-*trans* conformation. The bridging chain, –S-(CH_2_)_4_-S–, is almost planar (r.m.s. deviation for all non-H atoms: 0.056 Å) and its mean plane forms dihedral angles of 16.60 (7) and 5.80 (2)° with the aromatic rings. In the crystal, the mol­ecules are linked by weak C—H⋯O inter­actions into chains with graph-set notation *C*(14) along [0 0 1]. The crystal studied was a racemic twin, the ratio of the twin components being 0.27 (9):0.73 (9).

## Related literature

For the potential of coordination polymers based on multi­topic bridging ligands and metal centers as functional mat­erials, see: Guo *et al.* (2002 ▶); Melcer *et al.* (2001 ▶). For the use of flexible ligands in such structures, see: Bu *et al.* (2001 ▶); Withersby *et al.* (1997 ▶). For our studies on the synthesis and structural characterization of divalent sulfur compounds, see: Brito *et al.* (2004 ▶, 2005 ▶, 2006 ▶). For a related compound, see: Awaleh *et al.* (2005 ▶). For graph-set notation of hydrogen bonds, see: Bernstein *et al.* (1995 ▶).
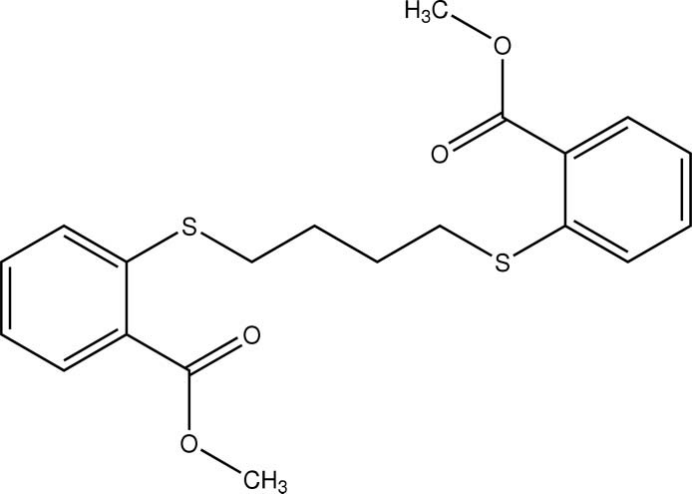


## Experimental

### 

#### Crystal data

C_20_H_22_O_4_S_2_
*M* *_r_* = 390.5
Orthorhombic, 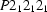

*a* = 7.4027 (7) Å
*b* = 14.2976 (11) Å
*c* = 17.7396 (13) Å
*V* = 1877.6 (3) Å^3^
*Z* = 4
Mo *K*α radiation
μ = 0.31 mm^−1^
*T* = 173 K
0.28 × 0.25 × 0.23 mm

#### Data collection

STOE IPDS II two-circle-diffractometer
Absorption correction: multi-scan (*MULABS*; Spek, 2003 ▶; Blessing, 1995 ▶) *T*_min_ = 0.919, *T*_max_ = 0.933
8050 measured reflections
3437 independent reflections
2244 reflections with *I* > 2σ(*I*)
*R*_int_ = 0.069

#### Refinement

*R*[*F* ^2^ > 2σ(*F* ^2^)] = 0.039
*wR*(*F* ^2^) = 0.065
*S* = 0.73
3437 reflections
239 parameters
H-atom parameters constrained
Δρ_max_ = 0.16 e Å^−3^
Δρ_min_ = −0.27 e Å^−3^
Absolute structure: Flack (1983 ▶), 1430 Friedel pairs
Flack parameter: 0.27 (9)

### 

Data collection: *X-AREA* (Stoe & Cie, 2001 ▶); cell refinement: *X-AREA*; data reduction: *X-AREA*; program(s) used to solve structure: *SHELXS97* (Sheldrick, 2008 ▶); program(s) used to refine structure: *SHELXL97* (Sheldrick, 2008 ▶); molecular graphics: *XP* (Sheldrick, 2008 ▶) and *PLATON* (Spek, 2003 ▶; software used to prepare material for publication: *WinGX* (Farrugia, 1999 ▶).

## Supplementary Material

Crystal structure: contains datablocks global, I. DOI: 10.1107/S1600536809042846/om2290sup1.cif

Structure factors: contains datablocks I. DOI: 10.1107/S1600536809042846/om2290Isup2.hkl

Additional supplementary materials:  crystallographic information; 3D view; checkCIF report

## Figures and Tables

**Table 1:** Hydrogen-bond geometry (Å, °)

*D*—H⋯*A*	*D*—H	H⋯*A*	*D*⋯*A*	*D*—H⋯*A*
C15—H15⋯O3^i^	0.95	2.45	3.123 (5)	127

Symmetry code: (i) 
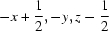
.

**Fig. 1.**
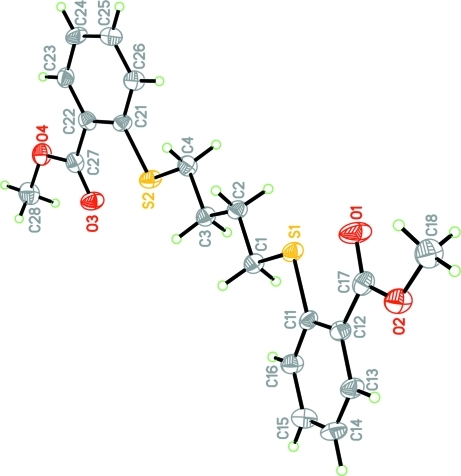
A view of the molecular structure of the title compound, with the atom-numbering scheme. Displacement ellipsoids are drawn at the 50% probability level and H atoms are shown as small spheres of arbitrary radii.

**Fig. 2.**
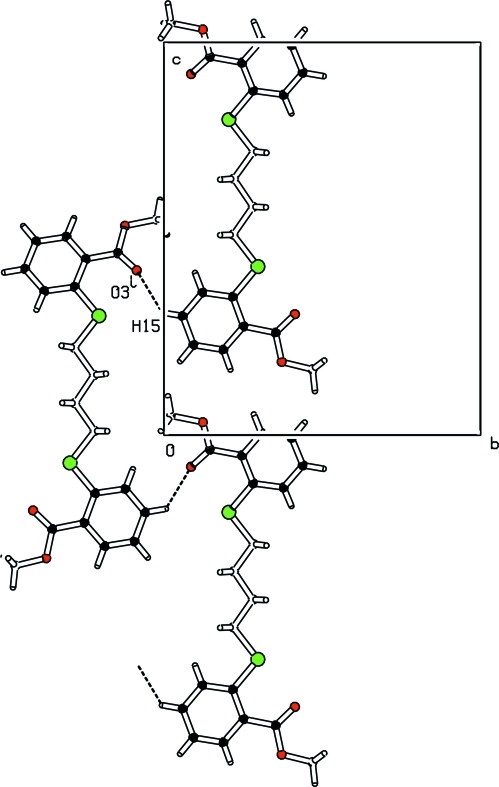
Part of the crystal structure of the title compound viewed along the a axis, showing the formation of a C(14) chain along the [0 0 1] direction. [Symmetry code for O3: 1/2 - x, -y, -1/2 + z.]

## supplementary crystallographic information

### Comment

In recent years, the rational design of coordination polymers based on multitopic bridging ligands and metal centers represents one of the most rapidly developing fields owing to their potential as functional materials (Guo *et al.*, 2002; Melcer *et al.*, 2001). The use of flexible ligands in such studies has attracted increasing attention because the flexibility and conformational freedom of such ligands offer the possibility for the construction of diverse frameworks with tailored properties and functions (Bu *et al.*, 2001; Withersby *et al.*, 1997).

The structure of the title compound is described here as part of our work involving the study of the synthesis and structural characterization of divalent-sulfur compounds (Brito *et al.*, 2004, 2005, 2006). The title compound is a longer analogue of 2,2'-dithiodibenzoate with the two benzoate units interconnected by a flexible –S-(CH_2_)_4_-S– bridge. The bridging chain moiety, –S-(CH_2_)_4_-S– is almost planar (r.m.s. deviation for all non-H atoms: 0.056 Å). Its mean plane forms a dihedral angle of 16.60 (7) and 5.80 (2)° with the aromatic rings. The C(*sp*^2^)-S bond lengths [1.780 (3), 1.773 (3) Å] are significantly shorter than the C(*sp*^3^)-S [1.821 (3), 1.822 (3) Å] bond lengths due to *p*-π conjugation, similar to that observed in 1,6-Bis(phenylsulfanyl)hexane (Awaleh *et al.*, 2005). The torsion angles in the aliphatic segment of the title compound are all *trans*, indicating that the molecule is in the fully extended conformation. The supramolecular structure of the title compound depends solely upon C—H···O hydrogen bonds: there are no significant S···S nor S···O contacts present in the structure and C—H···π (arene) hydrogen bonds and aromatic π···π stacking interactions are also absent. Atom C15 in the molecule at (*x*,*y*,*z*) acts as a hydrogen-bond donor to the carbonyl O3 atom in the molecule at (-*x* + 1/2,-*y*,*z* - 1/2), thereby generating a C(14) chain (Bernstein *et al.*, 1995) running in the [0 0 1] direction (Figure 2). The molecular stucture is stabilized by two C—H···O intramolecular hydrogen bonds (Table 1).

### Experimental

The title compound was synthesized as follows: a solution of 1,4-dibromobutene (1.08 g, 5 mmol) in ethanol (10 ml) was added dropwise to a mixture of methyl thiosalicylate (1.85 g, 11 mmol), KOH (0.617 g, 11 mmol) and ethanol (10 ml). The reaction mixture was stirred for 24 h at room temperature. The precipitate was filtered off, washed with water.Yield 80%; m.p. 393–396 K. FT—IR (KBr pellets, cm^-1^): ν (w, C—H of CH_3 _(*asym*.)) 2984, ν (w, C—H of CH_3_(*sym*.)) 2842, ν (w, C—H (aliphatic chain, *sym*) 2947, ν (w, C—H (chain aliphatic, *asym*)2919, ν (s, C=O) 1703, ν (s, C—H disubstitution 1,2) 1429, ν (s, O—C(CH_3_) 1252, ν (w, C—S) 749. Analysis calculated for C_20_H_22_O_4_S_2, _C 61.51, H 5.68, S 16.42%; found C 61.47, H, 5.72, S 15.32. Crystals suitable for single-crystals X-ray analysis were obtained by recrystallization from an acetonitrile solution.

### Refinement

Hydrogen atoms were located in a difference Fourier map but the were included in calculated positions [C—H = 0.95 - 0.99 Å] and refined as riding [*U*_iso_(H) = 1.2*U*_eq_(C) or 1.5*U*_eq_(C_methyl_)]. The crystal was refined as an racemic twin with a ratio of the twin components of 0.27 (9)/0.73 (9).

### Crystal data

**Table:**

C_20_H_22_O_4_S_2_	*F*(000) = 824
*M**_r_* = 390.5	*D*_x_ = 1.381 Mg m^−^^3^
Orthorhombic, *P*2_1_2_1_2_1_	Mo *K*α radiation, λ = 0.71073 Å
Hall symbol: P 2ac 2ab	Cell parameters from 4482 reflections
*a* = 7.4027 (7) Å	θ = 3.4–25.9°
*b* = 14.2976 (11) Å	µ = 0.31 mm^−^^1^
*c* = 17.7396 (13) Å	*T* = 173 K
*V* = 1877.6 (3) Å^3^	Block, colourless
*Z* = 4	0.28 × 0.25 × 0.23 mm

### Data collection

**Table:**

STOE IPDS II two-circle-diffractometer	2244 reflections with *I* > 2σ(*I*)
graphite	*R*_int_ = 0.069
ω scans	θ_max_ = 25.6°, θ_min_ = 3.3°
Absorption correction: multi-scan (*MULABS*; Spek, 2003; Blessing, 1995)	*h* = −8→7
*T*_min_ = 0.919, *T*_max_ = 0.933	*k* = −17→13
8050 measured reflections	*l* = −21→20
3437 independent reflections	

### Refinement

**Table:**

Refinement on *F*^2^	Hydrogen site location: inferred from neighbouring sites
Least-squares matrix: full	H-atom parameters constrained
*R*[*F*^2^ > 2σ(*F*^2^)] = 0.039	*w* = 1/[σ^2^(*F*_o_^2^) + (0.0001*P*)^2^] where *P* = (*F*_o_^2^ + 2*F*_c_^2^)/3
*wR*(*F*^2^) = 0.065	(Δ/σ)_max_ < 0.001
*S* = 0.73	Δρ_max_ = 0.16 e Å^−^^3^
3437 reflections	Δρ_min_ = −0.27 e Å^−^^3^
239 parameters	Extinction correction: *SHELXL*
0 restraints	Extinction coefficient: 0.0030 (3)
Primary atom site location: structure-invariant direct methods	Absolute structure: Flack (1983), 1430 Friedel pairs
Secondary atom site location: difference Fourier map	Flack parameter: 0.27 (9)

### Special details

**Table:**

Geometry. All e.s.d.'s (except the e.s.d. in the dihedral angle between two l.s. planes) are estimated using the full covariance matrix. The cell e.s.d.'s are taken into account individually in the estimation of e.s.d.'s in distances, angles and torsion angles; correlations between e.s.d.'s in cell parameters are only used when they are defined by crystal symmetry. An approximate (isotropic) treatment of cell e.s.d.'s is used for estimating e.s.d.'s involving l.s. planes.
Refinement. Refinement of *F*^2^ against ALL reflections. The weighted *R*-factor *wR* and goodness of fit *S* are based on *F*^2^, conventional *R*-factors *R* are based on *F*, with *F* set to zero for negative *F*^2^. The threshold expression of *F*^2^ > σ(*F*^2^) is used only for calculating *R*-factors(gt) *etc*. and is not relevant to the choice of reflections for refinement. *R*-factors based on *F*^2^ are statistically about twice as large as those based on *F*, and *R*- factors based on ALL data will be even larger.

### Fractional atomic coordinates and isotropic or equivalent isotropic displacement parameters (Å^2^)

**Table:**

	*x*	*y*	*z*	*U*_iso_*/*U*_eq_	
S1	0.26565 (13)	0.29690 (6)	0.42902 (3)	0.0257 (2)	
S2	0.30831 (13)	0.20540 (6)	0.80343 (4)	0.0292 (2)	
O1	0.2673 (5)	0.41362 (15)	0.30785 (10)	0.0449 (8)	
O2	0.2727 (4)	0.37231 (15)	0.18638 (9)	0.0340 (6)	
O3	0.3720 (4)	0.08601 (17)	0.91935 (11)	0.0359 (7)	
O4	0.4816 (4)	0.12269 (18)	1.03289 (10)	0.0369 (7)	
C1	0.2650 (6)	0.2191 (2)	0.51027 (13)	0.0249 (8)	
H1A	0.1554	0.1794	0.5099	0.03*	
H1B	0.3725	0.1779	0.5092	0.03*	
C2	0.2678 (6)	0.2809 (2)	0.58124 (13)	0.0264 (8)	
H2A	0.3754	0.322	0.5799	0.032*	
H2B	0.1591	0.3211	0.5819	0.032*	
C3	0.2725 (6)	0.2212 (2)	0.65279 (13)	0.0261 (9)	
H3A	0.1607	0.1834	0.6561	0.031*	
H3B	0.3766	0.1779	0.6506	0.031*	
C4	0.2885 (6)	0.2832 (2)	0.72250 (13)	0.0280 (9)	
H4A	0.1803	0.3234	0.7276	0.034*	
H4B	0.3964	0.3238	0.7187	0.034*	
C11	0.2723 (5)	0.2174 (2)	0.35190 (13)	0.0232 (9)	
C12	0.2798 (6)	0.2517 (2)	0.27679 (15)	0.0244 (9)	
C13	0.2887 (5)	0.1872 (2)	0.21715 (14)	0.0288 (9)	
H13	0.2957	0.2098	0.1669	0.035*	
C14	0.2875 (6)	0.0934 (2)	0.22956 (15)	0.0350 (10)	
H14	0.2954	0.0511	0.1883	0.042*	
C15	0.2747 (6)	0.0593 (2)	0.30303 (15)	0.0339 (9)	
H15	0.2696	−0.0061	0.3121	0.041*	
C16	0.2695 (6)	0.1219 (2)	0.36288 (14)	0.0282 (9)	
H16	0.2638	0.0983	0.4129	0.034*	
C17	0.2741 (6)	0.3527 (2)	0.26050 (14)	0.0276 (9)	
C18	0.2495 (7)	0.4698 (2)	0.16715 (15)	0.0389 (10)	
H18A	0.3472	0.5066	0.1897	0.058*	
H18B	0.1331	0.4919	0.1864	0.058*	
H18C	0.2525	0.477	0.1122	0.058*	
C21	0.3729 (5)	0.2822 (3)	0.87713 (15)	0.0249 (9)	
C22	0.4253 (6)	0.2465 (3)	0.94829 (16)	0.0266 (9)	
C23	0.4802 (5)	0.3083 (3)	1.00471 (16)	0.0308 (9)	
H23	0.5158	0.2841	1.0523	0.037*	
C24	0.4840 (6)	0.4036 (3)	0.99282 (17)	0.0349 (10)	
H24	0.5217	0.4446	1.0319	0.042*	
C25	0.4321 (5)	0.4390 (2)	0.92306 (17)	0.0319 (9)	
H25	0.435	0.5045	0.914	0.038*	
C26	0.3758 (6)	0.3778 (3)	0.86655 (16)	0.0294 (10)	
H26	0.3385	0.4027	0.8194	0.035*	
C27	0.4227 (5)	0.1458 (3)	0.96319 (16)	0.0287 (9)	
C28	0.4834 (6)	0.0230 (3)	1.04913 (17)	0.0392 (11)	
H28A	0.5551	−0.0095	1.0108	0.059*	
H28B	0.3594	−0.001	1.0485	0.059*	
H28C	0.5368	0.0125	1.099	0.059*	

### Atomic displacement parameters (Å^2^)

**Table:**

	*U*^11^	*U*^22^	*U*^33^	*U*^12^	*U*^13^	*U*^23^
S1	0.0329 (6)	0.0275 (4)	0.0166 (3)	−0.0011 (5)	−0.0012 (4)	−0.0023 (3)
S2	0.0443 (6)	0.0259 (5)	0.0175 (3)	−0.0023 (5)	−0.0008 (3)	0.0001 (4)
O1	0.084 (3)	0.0299 (14)	0.0212 (9)	−0.0002 (16)	−0.0042 (15)	−0.0037 (9)
O2	0.0500 (19)	0.0330 (13)	0.0189 (9)	0.0006 (14)	0.0028 (12)	0.0030 (9)
O3	0.053 (2)	0.0289 (15)	0.0253 (11)	−0.0018 (13)	−0.0031 (12)	−0.0013 (10)
O4	0.047 (2)	0.0387 (16)	0.0253 (10)	0.0049 (14)	−0.0123 (11)	0.0070 (11)
C1	0.028 (2)	0.0275 (19)	0.0195 (12)	−0.0018 (19)	0.0011 (14)	0.0010 (12)
C2	0.033 (2)	0.027 (2)	0.0193 (12)	0.0011 (19)	−0.0009 (16)	−0.0032 (12)
C3	0.029 (3)	0.027 (2)	0.0224 (14)	−0.002 (2)	−0.0004 (14)	0.0007 (11)
C4	0.035 (2)	0.031 (2)	0.0182 (12)	0.005 (2)	−0.0012 (14)	0.0028 (13)
C11	0.019 (2)	0.032 (2)	0.0186 (13)	0.001 (2)	−0.0006 (14)	−0.0067 (12)
C12	0.026 (2)	0.0257 (18)	0.0211 (13)	0.0007 (18)	0.0009 (15)	−0.0021 (12)
C13	0.033 (3)	0.034 (2)	0.0190 (12)	0.001 (2)	0.0014 (14)	−0.0066 (13)
C14	0.045 (3)	0.034 (2)	0.0265 (14)	0.002 (2)	−0.0003 (18)	−0.0135 (14)
C15	0.039 (3)	0.0284 (18)	0.0339 (14)	0.0011 (19)	0.0039 (19)	−0.0061 (13)
C16	0.039 (3)	0.0237 (19)	0.0223 (13)	0.000 (2)	−0.0014 (16)	0.0005 (12)
C17	0.028 (3)	0.034 (2)	0.0207 (14)	−0.003 (2)	−0.0004 (16)	0.0007 (13)
C18	0.049 (3)	0.038 (2)	0.0294 (14)	−0.002 (2)	−0.0001 (18)	0.0080 (13)
C21	0.029 (2)	0.027 (2)	0.0190 (13)	0.0006 (18)	0.0024 (13)	−0.0059 (14)
C22	0.026 (3)	0.030 (2)	0.0235 (16)	0.0050 (17)	0.0031 (14)	0.0010 (13)
C23	0.028 (2)	0.036 (2)	0.0275 (15)	0.0040 (18)	−0.0051 (15)	−0.0031 (17)
C24	0.039 (3)	0.036 (2)	0.0298 (16)	−0.0001 (19)	−0.0028 (17)	−0.0144 (17)
C25	0.035 (3)	0.026 (2)	0.0343 (16)	−0.0007 (17)	0.0052 (19)	−0.0028 (16)
C26	0.038 (3)	0.029 (2)	0.0214 (14)	−0.0001 (19)	0.0012 (15)	0.0031 (15)
C27	0.030 (3)	0.036 (2)	0.0202 (14)	0.005 (2)	0.0016 (15)	0.0023 (15)
C28	0.050 (3)	0.035 (2)	0.0326 (18)	0.005 (2)	−0.0053 (17)	0.0124 (15)

### Geometric parameters (Å, °)

**Table:**

S1—C11	1.780 (3)	C13—C14	1.360 (5)
S1—C1	1.821 (3)	C13—H13	0.95
S2—C21	1.773 (3)	C14—C15	1.395 (4)
S2—C4	1.822 (3)	C14—H14	0.95
O1—C17	1.211 (4)	C15—C16	1.389 (4)
O2—C17	1.344 (3)	C15—H15	0.95
O2—C18	1.445 (4)	C16—H16	0.95
O3—C27	1.215 (4)	C18—H18A	0.98
O4—C27	1.352 (4)	C18—H18B	0.98
O4—C28	1.454 (4)	C18—H18C	0.98
C1—C2	1.538 (4)	C21—C26	1.380 (5)
C1—H1A	0.99	C21—C22	1.416 (4)
C1—H1B	0.99	C22—C23	1.395 (4)
C2—C3	1.529 (3)	C22—C27	1.465 (5)
C2—H2A	0.99	C23—C24	1.379 (5)
C2—H2B	0.99	C23—H23	0.95
C3—C4	1.526 (4)	C24—C25	1.392 (4)
C3—H3A	0.99	C24—H24	0.95
C3—H3B	0.99	C25—C26	1.395 (5)
C4—H4A	0.99	C25—H25	0.95
C4—H4B	0.99	C26—H26	0.95
C11—C16	1.379 (5)	C28—H28A	0.98
C11—C12	1.421 (4)	C28—H28B	0.98
C12—C13	1.404 (4)	C28—H28C	0.98
C12—C17	1.474 (4)		
			
C11—S1—C1	102.59 (14)	C16—C15—H15	120.3
C21—S2—C4	102.96 (15)	C14—C15—H15	120.3
C17—O2—C18	115.6 (2)	C11—C16—C15	122.0 (3)
C27—O4—C28	115.1 (3)	C11—C16—H16	119
C2—C1—S1	107.3 (2)	C15—C16—H16	119
C2—C1—H1A	110.3	O1—C17—O2	121.9 (3)
S1—C1—H1A	110.3	O1—C17—C12	124.8 (2)
C2—C1—H1B	110.3	O2—C17—C12	113.3 (3)
S1—C1—H1B	110.3	O2—C18—H18A	109.5
H1A—C1—H1B	108.5	O2—C18—H18B	109.5
C3—C2—C1	111.1 (2)	H18A—C18—H18B	109.5
C3—C2—H2A	109.4	O2—C18—H18C	109.5
C1—C2—H2A	109.4	H18A—C18—H18C	109.5
C3—C2—H2B	109.4	H18B—C18—H18C	109.5
C1—C2—H2B	109.4	C26—C21—C22	118.3 (3)
H2A—C2—H2B	108	C26—C21—S2	121.2 (2)
C4—C3—C2	110.5 (2)	C22—C21—S2	120.5 (3)
C4—C3—H3A	109.5	C23—C22—C21	119.4 (3)
C2—C3—H3A	109.5	C23—C22—C27	119.8 (3)
C4—C3—H3B	109.5	C21—C22—C27	120.8 (3)
C2—C3—H3B	109.5	C24—C23—C22	121.4 (3)
H3A—C3—H3B	108.1	C24—C23—H23	119.3
C3—C4—S2	106.9 (2)	C22—C23—H23	119.3
C3—C4—H4A	110.3	C23—C24—C25	119.4 (3)
S2—C4—H4A	110.3	C23—C24—H24	120.3
C3—C4—H4B	110.3	C25—C24—H24	120.3
S2—C4—H4B	110.3	C24—C25—C26	119.5 (3)
H4A—C4—H4B	108.6	C24—C25—H25	120.2
C16—C11—C12	118.3 (3)	C26—C25—H25	120.2
C16—C11—S1	121.6 (2)	C21—C26—C25	121.9 (3)
C12—C11—S1	120.1 (3)	C21—C26—H26	119
C13—C12—C11	118.8 (3)	C25—C26—H26	119
C13—C12—C17	119.8 (3)	O3—C27—O4	120.9 (3)
C11—C12—C17	121.4 (3)	O3—C27—C22	125.5 (3)
C14—C13—C12	121.7 (3)	O4—C27—C22	113.6 (3)
C14—C13—H13	119.2	O4—C28—H28A	109.5
C12—C13—H13	119.2	O4—C28—H28B	109.5
C13—C14—C15	119.8 (3)	H28A—C28—H28B	109.5
C13—C14—H14	120.1	O4—C28—H28C	109.5
C15—C14—H14	120.1	H28A—C28—H28C	109.5
C16—C15—C14	119.4 (3)	H28B—C28—H28C	109.5
			
C11—S1—C1—C2	177.5 (3)	C13—C12—C17—O2	2.0 (6)
S1—C1—C2—C3	−178.5 (3)	C11—C12—C17—O2	−176.6 (4)
C1—C2—C3—C4	176.1 (4)	C4—S2—C21—C26	7.4 (4)
C2—C3—C4—S2	−176.2 (3)	C4—S2—C21—C22	−171.7 (3)
C21—S2—C4—C3	168.3 (3)	C26—C21—C22—C23	−0.9 (6)
C1—S1—C11—C16	2.2 (4)	S2—C21—C22—C23	178.2 (3)
C1—S1—C11—C12	−178.1 (4)	C26—C21—C22—C27	179.2 (4)
C16—C11—C12—C13	−1.6 (7)	S2—C21—C22—C27	−1.6 (5)
S1—C11—C12—C13	178.7 (3)	C21—C22—C23—C24	0.3 (6)
C16—C11—C12—C17	177.0 (4)	C27—C22—C23—C24	−179.9 (4)
S1—C11—C12—C17	−2.7 (6)	C22—C23—C24—C25	−0.1 (6)
C11—C12—C13—C14	1.0 (7)	C23—C24—C25—C26	0.5 (6)
C17—C12—C13—C14	−177.6 (4)	C22—C21—C26—C25	1.3 (6)
C12—C13—C14—C15	0.9 (7)	S2—C21—C26—C25	−177.8 (3)
C13—C14—C15—C16	−2.2 (7)	C24—C25—C26—C21	−1.2 (6)
C12—C11—C16—C15	0.3 (7)	C28—O4—C27—O3	1.6 (5)
S1—C11—C16—C15	−180.0 (3)	C28—O4—C27—C22	−179.1 (3)
C14—C15—C16—C11	1.6 (7)	C23—C22—C27—O3	177.5 (3)
C18—O2—C17—O1	−4.3 (6)	C21—C22—C27—O3	−2.7 (7)
C18—O2—C17—C12	174.2 (4)	C23—C22—C27—O4	−1.8 (6)
C13—C12—C17—O1	−179.6 (4)	C21—C22—C27—O4	178.1 (3)
C11—C12—C17—O1	1.8 (7)		

### Hydrogen-bond geometry (Å, °)

**Table:**

*D*—H···*A*	*D*—H	H···*A*	*D*···*A*	*D*—H···*A*
C13—H13···O2	0.95	2.36	2.705 (4)	101
C23—H23···O4	0.95	2.35	2.701 (5)	101
C15—H15···O3^i^	0.95	2.45	3.123 (5)	127

Symmetry codes: (i) −*x*+1/2, −*y*, *z*−1/2.
